# Study on Scattering and Absorption Properties of Quantum-Dot-Converted Elements for Light-Emitting Diodes Using Finite-Difference Time-Domain Method

**DOI:** 10.3390/ma10111264

**Published:** 2017-11-03

**Authors:** Jiasheng Li, Yong Tang, Zongtao Li, Xinrui Ding, Dong Yuan, Binhai Yu

**Affiliations:** 1 Engineering Research Center of Green Manufacturing for Energy-Saving and New-Energy Technology, South China University of Technology, Guangzhou 510640, China; jiasli@foxmail.com (J.L.); ytang@scut.edu.cn (Y.T.); bhaiyu@163.com (B.Y.); 2 Foshan Nationstar Optoelectronics Company Ltd., Foshan 528000, China; 3 Department of Mechanical Engineering, University of California, Berkeley, CA 94720-5800, USA; xrding@berkeley.edu; 4 South China Academy of Advanced Optoelectronics, South China Normal University, Guangzhou 510640, China; yuandong@scnu.edu.cn

**Keywords:** light-emitting diodes, photoluminescence, quantum dots, finite-difference time-domain

## Abstract

CdSe/ZnS quantum-dot-converted elements (QDCEs) are good candidates for substituting rare-earth phosphor-converted elements (PCEs) in white light-emitting diodes (LEDs); however, studies on their scattering and absorption properties are scarce, suppressing further increment in the optical and thermal performance of quantum-dot-converted LEDs. Therefore, we introduce the finite-difference time-domain (FDTD) method to achieve the critical optical parameters of QDCEs when used in white LEDs; their scattering cross-section (coefficient), absorption cross-section (coefficient), and scattering phase distributions are presented and compared with those of traditional YAG phosphor-converted elements (PCEs) at varying particle size and concentration. At a commonly used concentration ($<50\;\mathrm{mg}/{\mathrm{cm}}^{3}$), QDCEs exhibit stronger absorption (tens of millimeters, even for green-to-red-wavelength light) and weaker scattering ($<1{\;\mathrm{mm}}^{-1}$) compared to PCEs; the reabsorption, total internal reflection, angular uniformity, and thermal quenching would be more significant concerns for QDCEs. Therefore, the unique scattering and absorption properties of QDCEs should be considered when used in white LEDs. Furthermore, knowledge of these important optical parameters is helpful for beginning a theoretical study on quantum-dot-converted LEDs according to the ray tracing method.

## 1. Introduction

Solution-processed colloids attract a great deal of attention due to their tunable and color-saturated emission properties [1]. CdSe/ZnS (core/shell) quantum dots (QDs) have been widely studied and found to improve quantum yield and stability [2], and have become a promising down-conversion material for white light-emitting diodes (LEDs). However, QDs still pose challenges when used in white LEDs compared to traditional YAG phosphor down-conversion materials [3], such as low luminous efficacy and low thermal reliability [4]. Phosphor-converted elements (PCEs) that contain dispersed phosphors in a silicone matrix play an important role in the performance of LEDs [5], which is the same as that of quantum-dot-converted elements (QDCEs) (color-conversion elements with QDs embedded in silicone matrix). Recently, researchers have begun to experimentally study QDCEs in order to produce high-performance LEDs by embedding functional particles [6,7] and optimizing packaging structures [8,9,10]. The experimental results obtained in many of these studies indicate that QDCEs show different scattering and absorption properties to those of PCEs.

The scattering and absorption properties of PCEs are essential for the design of phosphor-converted LEDs. Sommer et al. found that the YAG phosphor particle size can greatly affect the angular homogeneity and radiant flux of LEDs due to the difference in their scattering functions [11,12]; moreover, they also revealed that the absorption properties of PCEs greatly affect their thermal load [13,14]. Alongside this research, Luo et al. have investigated the transmittance and reflection of PCEs with different concentration and particle size according to their scattering and absorption properties [15]. Besides, we indicated that PCEs with proper nitride and YAG phosphor proportion can gain a high optical power and color uniformity due to difference in phosphor scattering and absorption properties [16,17]. Together, these studies have provided a better understanding of the design of phosphor-converted LEDs. The scattering and absorption properties of PCEs are critical for the optical modeling of LEDs [16,18,19], which has motivated a great deal of theoretical study on PCE optimization [20,21,22] through use of the Monte Carlo bulk scattering model [23]. However, the scattering and absorption properties of QDCEs have not yet been investigated, and theoretical studies on quantum-dot-converted LEDs have been rarely reported. This is mainly because the Mie theory used for traditional PCE calculations can only obtain an accurate mathematical solution for a homogenous spherical particle [24]; it is an ideal method for spherical YAG phosphors, yet is unsuitable for commercial CdSe/ZnS QDs with heterogeneous structures. Previously, we have introduced the finite-difference time-domain (FDTD) method [25]—which is widely used in the study of nanostructures—to solve the optical properties of PCEs with non-spherical nitride phosphor particles; it is also expected to use this method for QDCEs.

In this study, we apply the FDTD method to achieve the scattering and absorption properties of QDCEs. These properties are critical for quantum-dot-converted LEDs, and are discussed and compared with those of PCEs in order to obtain a better understanding of the optical characteristics of QDCEs when used in white LEDs.

## 2. Method

Similar to the PCEs used in white LEDs, the scattering and absorption properties of QDCEs can be determined by the Monte Carlo bulk scattering model [23]. Three critical parameters are included: absorption coefficient, scattering coefficient, and scattering phase function. The absorption coefficient $\mu_{\mathrm{abs}}\left(\lambda \right)$ and scattering coefficient $\mu_{\mathrm{sca}}\left(\lambda \right)$ describe the absorption probability and scattering probability, respectively. These coefficients take the following forms [26]:

(1) $$\mu_{\mathrm{abs}}\left(\lambda \right)=\frac{c}{\bar{m}}\int \;f\left(D\right)C_{\mathrm{abs}}\left(D,\lambda \right)dD$$

(2) $$\mu_{\mathrm{sca}}\left(\lambda \right)=\frac{c}{\bar{m}}\int \;f\left(D\right)C_{\mathrm{sca}}\left(D,\lambda \right)dD$$

where $c/\bar{m}$ represents the particle density of the QDs; c is the QD concentration ($\mathrm{mg}/{\mathrm{cm}}^{3}$); D is the particle size (nm); λ is the wavelength (nm); f(D) is the QD particle size distribution function; $\bar{m}$ is the mean mass of QD (mg) in the QDCE, which can be calculated by integrating over f(D); $C_{\mathrm{abs}}\left(D,\lambda \right)$ and $C_{\mathrm{sca}}\left(D,\lambda \right)$ are the absorption cross-section and scattering cross-section of the QD, respectively, and they are defined as

(3) $$C_{\mathrm{abs}}\left(D,\lambda \right)=\frac{P_{\mathrm{abs}}\left(D,\lambda \right)}{P_{\mathrm{inc}}\left(\lambda \right)}=\frac{\int \;p_{\mathrm{tol}}\left(\theta ,D,\lambda \right)d\theta -\int \;p_{\mathrm{sca}}\left(\theta ,D,\lambda \right)d\theta }{P_{\mathrm{inc}}\left(\lambda \right)}$$

(4) $$C_{\mathrm{sca}}\left(D,\lambda \right)=\frac{P_{\mathrm{sca}}\left(D,\lambda \right)}{P_{\mathrm{inc}}\left(\lambda \right)}=\frac{\int \;P_{\mathrm{sca}}\left(\theta ,D,\lambda \right)d\theta }{P_{\mathrm{inc}}\left(\lambda \right)}$$

where $P_{\mathrm{inc}}\left(\lambda \right)$ is the incident irradiance of source ($W/m^{2})$; $P_{\mathrm{abs}}\left(D,\lambda \right)$ and $P_{\mathrm{sca}}\left(D,\lambda \right)$ are the absorption power and scattering power (W), respectively, when light propagates through the QD; $p_{\mathrm{tol}}\left(\theta ,D,\lambda \right)$ and $p_{\mathrm{sca}}\left(\theta ,D,\lambda \right)$ are the angular total power and scattering power (W), respectively. The scattering phase function which is used to describe the scattering energy distribution satisfies the normalization conditions [27], and can be written in the simplified form

(5) $$p\left(\theta ,\lambda \right)=\frac{\int \;f\left(D\right)(p_{\mathrm{sca}}\;\left(\theta ,D,\lambda \right)/P_{\mathrm{sca}}\;\left(D,\lambda \right)C_{\mathrm{sca}}\left(D,\lambda \right)dD}{\int \;f\left(D\right)C_{\mathrm{sca}}\left(D,\lambda \right)dD}$$

**Figure 1:**
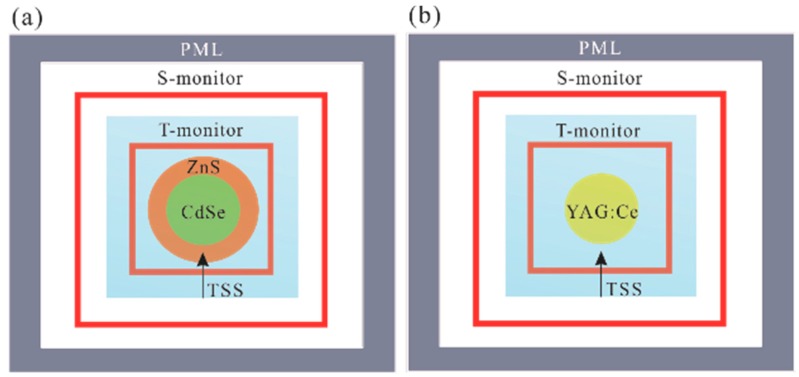
Finite-difference time-domain (FDTD) modeling of (**a**) CdSe/ZnS quantum dot (QD) and (**b**) YAG phosphor. PML: perfectly matched layer; TSS: total-field scattered-field source.

With Equations (1)–(5), $\mu_{\mathrm{abs}}\left(\lambda \right)$, $\mu_{\mathrm{sca}}\left(\lambda \right)$, and p(θ,λ) can be obtained once $p_{\mathrm{tol}}\left(\theta ,D,\lambda \right)$ and $p_{\mathrm{sca}}\left(\theta ,D,\lambda \right)$ are known. The FDTD method involves calculating the electromagnetic energy in discrete time and space intervals based on Maxwell’s equations, and is applied to find these values as shown in Figure 1. The commercial software of FDTD Solutions from Lumerical was used to perform the three-dimensional (3-D) FDTD simulations. The perfectly matched layer (PML) acts as the boundary condition for the 3-D FDTD region. The total-field scattered-field source (TSS) is used to divide the calculation region into the total field region (TFR) and the scattering field region (SFR). In the TFR, both the incident source electromagnetic energy and scattered electromagnetic energy are included, while only the scattered electromagnetic energy is calculated in the SFR. The T-monitor and S-monitor are used to collect the total and scattered electromagnetic energy, respectively. Therefore, the $p_{\mathrm{tol}}\left(\theta ,D,\lambda \right)$ and $p_{\mathrm{sca}}\left(\theta ,D,\lambda \right)$ can be found by integrating the Poynting vector [21] in the TFR and SFR, respectively. Both the CdSe/ZnS QD and YAG phosphor are modeled as spherical in shape, with the CdSe core size ranging from 2 to 6 nm in diameter and the shell size ranging from 0 molecular layers (MLs) to 10 MLs (1 ML is approximately 0.31 nm [2]), while the YAG phosphor size ranges from 1 to 25 μm in diameter. The CdSe core, ZnS shell, and YAG phosphor material have a wavelength-dependent complex refractive index [28], while the background has a refractive index of 1.54, assuming that the CdSe/ZnS and YAG phosphors are embedded in the same silicone matrix when used in white LEDs. To improve the accuracy of the simulation results, the mesh size is set to 0.005 nm for CdSe/ZnS QDs and 10 nm for YAG phosphors, due to their different minimum particle size (the mesh size is set as approximately two orders of magnitude smaller than their minimum particle size). The simulation time is set to a sufficiently large value to ensure energy convergence (<0.001%).

## 3. Results and Discussion

**Figure 2:**
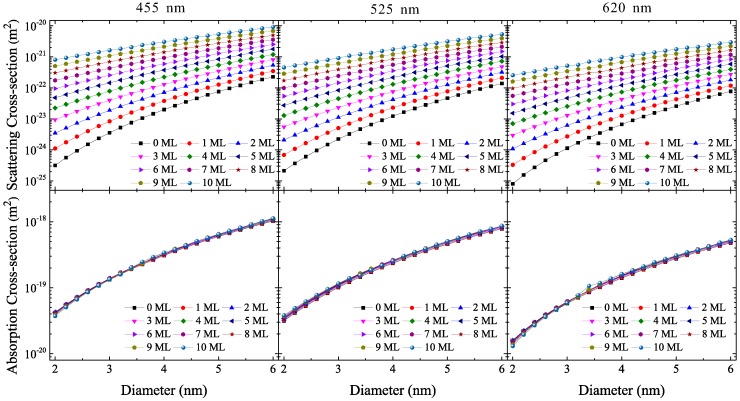
Scattering and absorption cross-sections of CdSe/ZnS quantum dots (QDs). (MLs: molecule layers).

The scattering and absorption cross-section of the CdSe/ZnS QD, $C_{\mathrm{qd}\_\mathrm{sca}}$ and $C_{\mathrm{qd}\_\mathrm{abs}}$, are shown in Figure 2 for varying ML numbers and three typical wavelengths: 455 nm (blue), 525 nm (green), 620 nm (red). As the core size increases, $C_{\mathrm{qd}\_\mathrm{sca}}$ and $C_{\mathrm{qd}\_\mathrm{abs}}$ also increase, resulting in stronger scattering and absorption ability. Moreover, as the number of MLs increases, $C_{\mathrm{qd}\_\mathrm{sca}}$ can increase by several orders of magnitude while $C_{\mathrm{qd}\_\mathrm{abs}}$ remains approximately constant. This is because the main contribution to the absorption comes from the CdSe core instead of the ZnS shell, although the ZnS shell with its high refractive index also contributes to the scattering. Despite $C_{\mathrm{qd}\_\mathrm{sca}}$ being more sensitive to the effect of QD size than $C_{\mathrm{qd}\_\mathrm{abs}}$, its value is 2–5 orders of magnitude smaller than that of $C_{\mathrm{qd}\_\mathrm{abs}}$ for a QD with the same core diameter and shell ML number, indicating that the absorption ability of the CdSe/ZnSe QD is significantly stronger than its scattering ability. $C_{\mathrm{qd}\_\mathrm{sca}}$ is notably smaller than $C_{\mathrm{qd}\_\mathrm{abs}}$ when the core diameter and the shell ML number decrease—a result that further suggests that smaller QD size leads to a larger difference between scattering and absorption ability. Both $C_{\mathrm{qd}\_\mathrm{sca}}$ and $C_{\mathrm{qd}\_\mathrm{abs}}$ show a slight decrease with increasing wavelength of incident light, and in addition to the effect of their complex refractive index, their small size relative to the wavelength also plays an important role because of the stronger diffraction effect that occurs when the CdSe/ZnS QD size and incident wavelength are comparable [29]. It should be noted that the variation in $C_{\mathrm{qd}\_\mathrm{abs}}$ for different wavelengths stays within a single order of magnitude, possibly promoting undesirable reabsorption in white LEDs [30].

The scattering and absorption cross-sections of the YAG phosphor, $C_{\mathrm{psr}\_\mathrm{sca}}$ and $C_{\mathrm{psr}\_\mathrm{abs}}$, are given in Figure S1 for comparison with the equivalent values for CdSe/ZnS QD. Similarly, $C_{\mathrm{psr}\_\mathrm{sca}}$ and $C_{\mathrm{psr}\_\mathrm{abs}}$ increase with increasing particle size, and their order of magnitude are consistent with those reported in previous studies [16,19]. It is reasonable that both $C_{\mathrm{psr}\_\mathrm{sca}}$ and $C_{\mathrm{psr}\_\mathrm{abs}}$ are several orders of magnitude larger than $C_{\mathrm{qd}\_\mathrm{sca}}$ and $C_{\mathrm{qd}\_\mathrm{abs}}$ due to the microscale particle size of YAG phosphors (as their cross-section is closely related to the projected area of the particles [31]). Therefore, undoubtedly, the scattering and absorption ability of a single YAG phosphor is much stronger than those of a single CdSe/ZnS QD due to their difference in size. However, it is interesting that $C_{\mathrm{psr}\_\mathrm{sca}}$ is 1–4 orders of magnitude larger than $C_{\mathrm{psr}\_\mathrm{abs}}$. This shows that the scattering ability of a YAG phosphor is much stronger than its absorption ability, which is the opposite situation to that of a CdSe/ZnS QD. The change in $C_{\mathrm{psr}\_\mathrm{abs}}$ for different wavelengths is over one order of magnitude (or two for red light), indicating that a single CdSe/ZnS QD particle (within one order of magnitude) has a higher probability of reabsorbing a down-converted photon compared with a single YAG phosphor particle.

**Figure 3:**
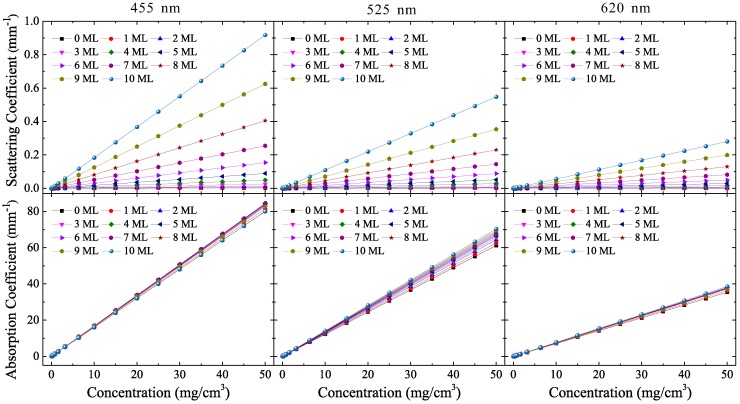
Scattering and absorption coefficients of quantum-dot-converted elements (QDCEs) with 3.2 nm CdSe/ZnS quantum dots.

From the cross-sections, the scattering and absorption coefficients of the QDCEs, $\mu_{\mathrm{qdce}\_\mathrm{sca}}$ and $\mu_{\mathrm{qdce}\_\mathrm{abs}}$, are shown in Figure 3. We have selected a core size of 3.2 nm (results for 4.2 nm and 5.2 nm are given in Figures S2 and S3, respectively, for reference) for discussion and comparison with YAG phosphors, because QDs of this size emit green-yellow light by down-conversion [2,32], which is the same as that emitted by YAG phosphors. For practical purposes, their concentration ranges from 0 to 50 $\mathrm{mg}/{\mathrm{cm}}^{3}$, which is the range generally adopted in the LED packaging process [6,7,8,9,10]. Similarly, the scattering and absorption coefficients of PCEs, $\mu_{\mathrm{pce}\_\mathrm{sca}}$ and $\mu_{\mathrm{pce}\_\mathrm{abs}}$, are shown in Figure S4. The particle size of the YAG phosphor is 13.7 μm, which is the same as that used in our previous studies [21], and their concentration ranges from 0 to 2000 $\mathrm{mg}/{\mathrm{cm}}^{3}$. Figure 3 shows that $\mu_{\mathrm{qdce}\_\mathrm{abs}}$ is hundreds of times larger than $\mu_{\mathrm{qdce}\_\mathrm{sca}}$, especially when QD size is small, resulting in an absorption probability that is much larger than the scattering probability inside the QDCE. Furthermore, $\mu_{\mathrm{qdce}\_\mathrm{sca}}$ is less than 1.0 ${\mathrm{mm}}^{-1}$, which is tens of times smaller than that of the commonly used PCE (~20 ${\mathrm{mm}}^{-1}$ [21]), as shown in Figure S4. Although $\mu_{\mathrm{qdce}\_\mathrm{sca}}$ can be increased by increasing the concentration above 50 $\mathrm{mg}/{\mathrm{cm}}^{3}$, $\mu_{\mathrm{qdce}\_\mathrm{abs}}$ for blue light simultaneously increases over 80 ${\mathrm{mm}}^{-1}$, as shown in Figure 3. It is concluded that such a high value of $\mu_{\mathrm{qdce}\_\mathrm{abs}}$ is trivial for generating white light, as the QDCE layer is several millimeters thick, so the blue light from the LED chips will be entirely absorbed. The fact that $\mu_{\mathrm{pce}\_\mathrm{abs}}$ is generally smaller than ~$10{\;\mathrm{mm}}^{-1}$ (as shown in Figure S4) also clarifies this issue. These results suggest that fewer scattering events occur in a QDCE than in a PCE. This contributes to the decrease in backward scattering loss, but it is also difficult to reduce the total internal reflection (TIR), increase the down-conversion efficiency, and balance light distributions to gain high color uniformity by utilizing the scattering effect [6]. In addition, it is interesting to note that the concentration of QDCEs is hundreds of times smaller than that of PCEs when $\mu_{\mathrm{qdce}\_\mathrm{abs}}$ and $\mu_{\mathrm{pce}\_\mathrm{abs}}$ are equal, likely due to the fact that fewer CdSe/ZnS QDs are needed in the packaging process [6,7,8,9,10] compared to YAG phosphors. However, this also means that the absorption power per concentration of QDCEs can be larger than that of PCEs, causing problematic thermal quenching as the quantum yield of CdSe/ZnS QDs is not ideal (100%). We consider these results to be useful in understanding that the stability of QDCEs is far lower than that of PCEs in white LEDs, especially in high current injection conditions (i.e., LEDs with high radiant power) [7]. It should be noted that $\mu_{\mathrm{qdce}\_\mathrm{abs}}$ also takes high values for green light and even red light, demonstrating very different behavior to $\mu_{\mathrm{pce}\_\mathrm{abs}}$ (smaller than $0.5{\;\mathrm{mm}}^{-1}$, as shown in Figure S4). This means that the QDCE has a higher probability of reabsorbing the converted light emission from QDs and other phosphors, such as nitride phosphor-emitted red light, potentially causing significant loss due to reabsorption [30].

**Figure 4:**
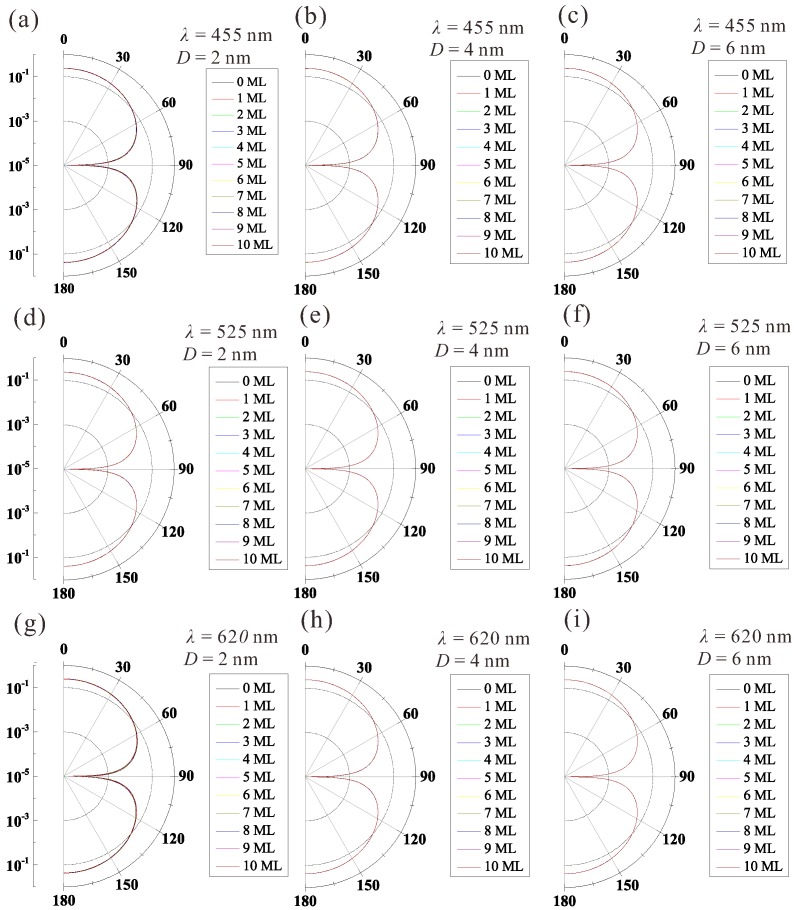
Scattering phase distributions of quantum-dot-converted elements (QDCEs) with varying diameter, *D*, and number of molecule layers (MLs) of CdSe/ZnS quantum dots (QDs). *λ*: wavelength of incident light.

The scattering phase functions of the QDCE, $p_{\mathrm{qdce}}$, are given in Figure 4. Their distributions clearly satisfy Rayleigh scattering and exhibit independence of wavelength and particle size because the particle size is smaller than the wavelength, while the PCE is considered to satisfy Mie scattering [19]. This means that the scattered light in QDCEs is more readily absorbed by LED chips, lead frames, and other packaging elements, as it has a higher probability of backward propagation compared with that of PCEs. However, we believe that this is not the major factor responsible for the decrease in luminous efficacy of quantum-dot-converted LEDs due to the low scattering probability in QDCEs discussed previously.

## 4. Conclusions

We have introduced the FDTD method to determine the scattering and absorption properties of CdSe/ZnS QDCEs, which have been compared with those of the traditional YAG PCEs. We find that the scattering coefficient of the QDCEs ($<1{\;\mathrm{mm}}^{-1}$) is tens of times lower than that of the PCEs, while the absorption coefficient of the QDCEs is so large that it can match that of the PCEs even at a concentration of QDCEs hundreds of times smaller than that of PCEs; moreover, the QDCE has a high absorption coefficient value for green-to-red-wavelength light comparable with that for blue-wavelength light. These results suggest that the scattering probability is lower (even can be neglected) while the absorption probability is significantly higher in QDCEs for different wavelengths, which is opposite to that of PCEs, potentially exhibiting a different effect on the optical and thermal performance in white LEDs compared to PCEs; the reabsorption, total internal reflection, angular uniformity, and thermal quenching would be more significant concerns for QDCEs. Therefore, the unique scattering and absorption properties of QDCEs should be considered when used in white LEDs. This study has provided a better understanding of scattering and absorption properties of QDCEs, which is important for the design and optimization of quantum-dot-converted LEDs. The study of such critical optical parameters also provides a foundation for theoretical study on quantum-dot-converted LEDs according to the ray tracing method.

In the future, we plan to use these fundamental parameters to study the effect of QDCE structures on the optical performance of LEDs. We believe that our findings will significantly contribute to the development of QDs in LED lighting applications.

## Supplementary Materials

The following are available online at www.mdpi.com/1996-1944/10/11/1264/s1. Figure S1: Scattering and absorption cross-sections of YAG phosphors. Figure S2: Scattering and absorption coefficients of quantum-dot-converted elements (QDCE) with 4.2 nm (5 molecule layers) CdSe/ZnS QDs. (MLs: molecular layers.). Figure S3: Scattering and absorption coefficients of quantum-dot-converted elements (QDCE) with 5.2 nm CdSe/ZnS quantum dots (QD). (MLs: molecular layers.). Figure S4: (a) Scattering and (b) absorption coefficients of phosphor-converted elements (PCE).
