# Supplementary material for: Study on Scattering and Absorption Properties of Quantum-Dot-Converted Elements for Light-Emitting Diodes Using Finite-Difference Time-Domain Method
Source: Materials (Basel). 2017 Nov 3;10(11):1264. doi: 10.3390/ma10111264 (PMC5706211; doi:10.3390/ma10111264)
Supplement: Supplementary file 1 [file materials-10-01264-s001.pdf]

Supplementary Materials:

Study on Scattering and Absorption Properties of Quantum-Dot-Converted Elements for Light-Emitting Diodes Using Finite-Difference Time-Domain Method

Jiasheng Li, Yong Tang, Zongtao Li, Xinrui Ding, Dong Yuan and Binhai Yu

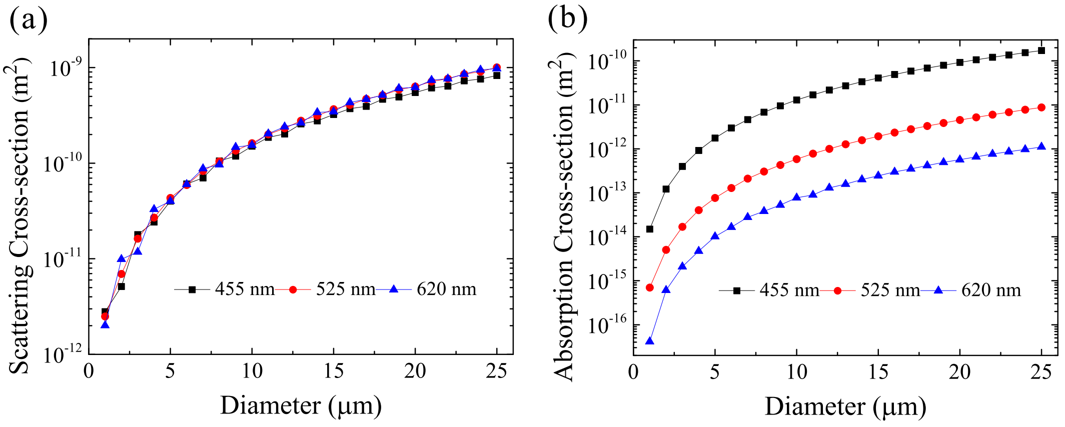

Figure S1. Scattering and absorption cross-sections of YAG phosphors.

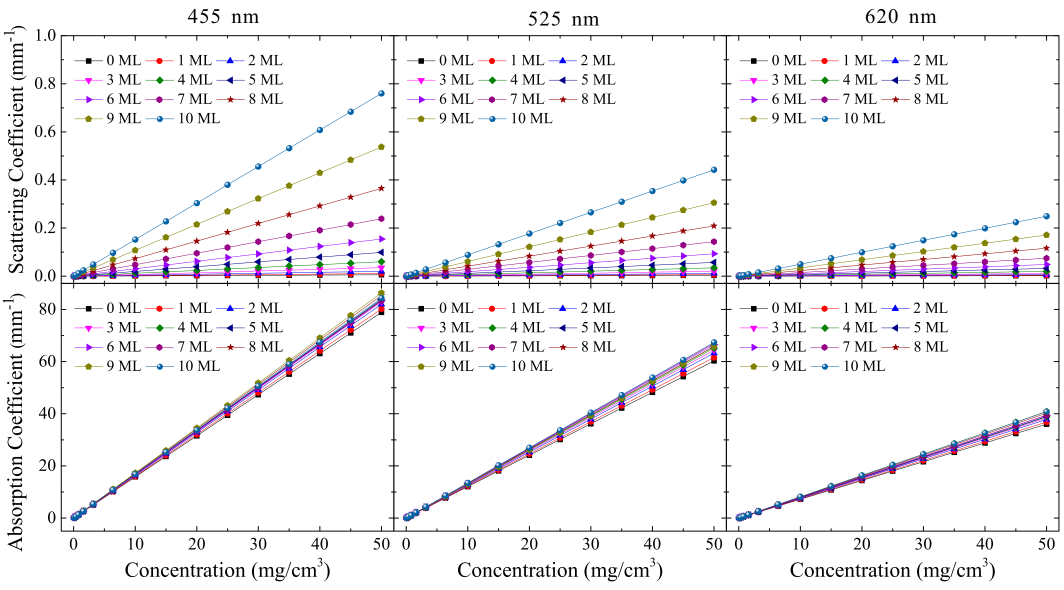

Figure S2. Scattering and absorption coefficients of quantum-dot-converted elements (QDCE) with 4.2 nm (5 molecule layers) CdSe/ZnS QDs. (MLs: molecular layers.)

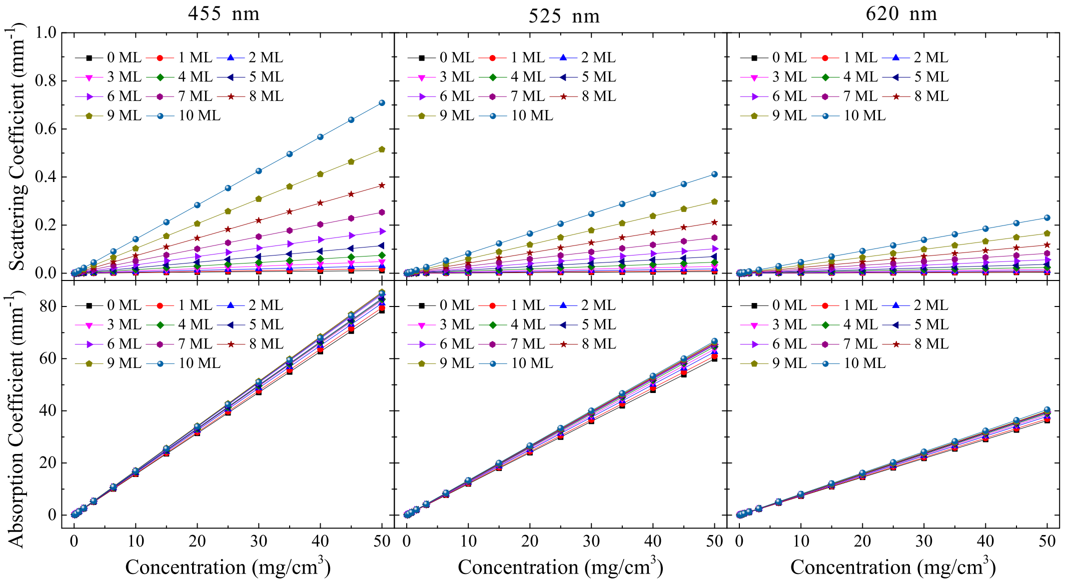

Figure S3. Scattering and absorption coefficients of quantum-dot-converted elements (QDCE) with 5.2 nm CdSe/ZnS quantum dots (QD). (MLs: molecular layers.)

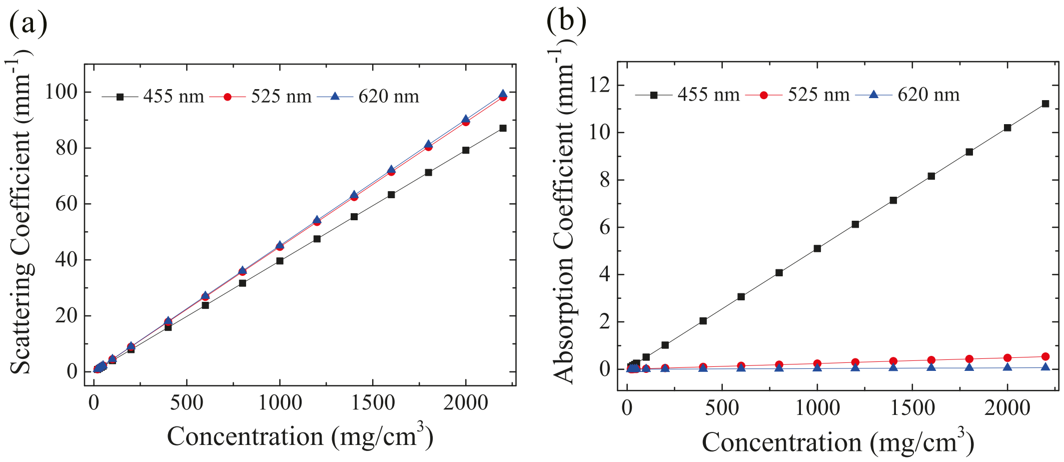

Figure S4. (a) Scattering and (b) absorption coefficients of phosphor-converted elements (PCE).
